# Supplementary material for: The Oldenburg Hearing Health Record (OHHR)
Source: Sci Data. 2025 Sep 17;12:1546. doi: 10.1038/s41597-025-05884-y (PMC12443984; doi:10.1038/s41597-025-05884-y)
Supplement: Supplementary file 1 — Supplementary Table 1 [file 41597_2025_5884_MOESM1_ESM.pdf]

Supplementary Table 1. Descriptive statistics of standardized measures in the OHHR and reference data from other studies

Assessment tool	Number of items	Measurement Intention	OHHR Sample (*Mean ± SD*; Range)	Published Norms (German population)
**Digit Triplet Test (DTT, Zokoll et al., 2012)[28]**	27-digit triplets	Speech Recognition Threshold in noise	*-4.6 dB ± 4.2;* -10.7 to 14.5 (dB)	Normal Hearing[28]:<br>*-9.3 ± 0.4* dB (headphones),<br>*-6.5 ± 0.4* dB (telephone)
**Göttingen Sentence Test (GÖSA, Kollmeier & Wesselkamp, 1997)[26]**	20 sentences	Speech Recognition Threshold in noise	*-1.5 dB ± 3.1*; -7.6 to 15.7 (dB)	(von Gablenz & Holube, 2017[75])<br>Young Normal Hearing:<br>*M ± SD: -4.8 ± 0.9* (dB); Range: -6.6 to -3 dB<br>(Hörtech Recommended[75])<br>*M ± SD : -6.2 ± 2.0* (dB); Range: -8.2 to -4.2 (dB)<br>(Thiele et al., 2012)[76]<br>Mild Hearing Loss: 0 ± 1-2 (dB),<br>Moderate Hearing Loss: 5 ± 6 (dB),<br>Severe Hearing Loss: >> 20 (dB)
**Dementia Detection Test (DemTect, Kalbe et al., 2004)[25]**	5 subtests	Cognitive impairment screening	*15.8 ± 2.3;* 7 to 18	Normal Hearing[25]:<br>Normal cognition: Scores ≥13,<br>Mild cognitive impairment: 9–12,<br>Suspected dementia: ≤8

**Vocabulary Size Test (WST, Schmidt & Metzler, 1992)[43]**	40 tasks	Indicator for crystallized intelligence	*31.5 ± 4.9*; 8 to 41	Normal Hearing[43]: Healthy young adults: *Mean 31-35* (depending on education)
**Short Form Health Survey (SF-12, Ware et al., 1996)[52] Physical Component Score**	6 items	Physical health score (Standardized)	*47.2* ± 9.2*; 18.7 to 62.9*	(Drixler et al., 2020)[77] Normal Hearing: *50 ± 10,*
**Short Form Health Survey (SF-12, Ware et al., 1996)[52] Mental Component Score**	6 items	Mental health score (Standardized)	*52.1* ± 8.9*; 21.8 to 70.1*	*50 ± 10*

Note: The SF-12 values are based on 568 participants due to 13 missing entries. The Physical Component Summary (PCS) and Mental Component Summary (MCS) scores were calculated separately from their respective 6 items each, out of the total 12 items. The mean, range, and standard deviation values for the other measures are based on the entire sample of 581 individuals.
